# Association of Oxidative Stress Biomarkers with Metabolic Parameters in Dairy Goats During the Periparturient Period

**DOI:** 10.3390/metabo15120790

**Published:** 2025-12-11

**Authors:** Giovanna Meli, Valentina Fumo, Wenning Chen, Giovanni Savoini, Guido Invernizzi

**Affiliations:** Dipartimento di Medicina Veterinaria e Scienze Animali, Università degli Studi di Milano, Via dell’Università 6, 26900 Lodi, Italywenning.chen@unimi.it (W.C.); giovanni.savoini@unimi.it (G.S.); guido.invernizzi@unimi.it (G.I.)

**Keywords:** dairy goats, oxidant status, ROS, antioxidant capacity, metabolic biomarkers

## Abstract

Objective: This study aimed at detecting changes in redox balance (reactive oxygen species (ROS), serum antioxidant capacity (SAC) and oxidative stress index (OSi)) during the peripartum period in dairy goats and evaluating the relationship between oxidative biomarkers (ROS, SAC and OSi) and selected metabolic biomarkers (total cholesterol, triglycerides, beta-hydroxybutyrate (BHB) and non-esterified fatty acids (NEFAs)). Materials and Methods: Blood samples were collected from 32 secondiparous Alpine dairy goats (average daily milk production: 3.93 ± 1.23 L) from the same commercial herd at dry-off, kidding and 30 days in milk (DIM). Results: Fatty acids at dry-off and BHB at kidding (rho: 0.56; *p* < 0.01) and 30 DIM (rho: 0.54; *p* < 0.01) were positively correlated, suggesting a potential role of pre-partum NEFA concentrations on the metabolic status of dairy goats during early lactation. Parturition was associated with the highest values of ROS (183.13 ± 6.99 Carr. U; *p* < 0.05) and OSi (0.50 ± 0.03 Carr. U/umol HCLO/mL; *p* < 0.05) probably due to the stress typical of this period. As expected, OSi exhibited a positive correlation with ROS (rho: 0.405, *p* < 0.01) and a negative correlation with SAC (rho: −0.707, *p* < 0.01). Furthermore, NEFAs showed a tendency to be positively correlated with ROS (rho: 0.191 *p* < 0.06) and were positively correlated with OSi (rho: 0.219 *p* < 0.03), suggesting a potential role of this metabolic parameter on oxidant status. Conclusions: The knowledge of the interplay between oxidative stress and metabolic changes during the peripartum period could potentially facilitate the development of strategies for the early detection and management of metabolic disorders in dairy goats.

## 1. Introduction

Transition from late pregnancy to early lactation represents an important period in the life of dairy animals, marked by significant alterations in both metabolic and immune functions. These changes increase the susceptibility to the onset of metabolic and infectious diseases [1,2]. Dairy goats during the periparturient period commonly experience a negative energy balance (NEB) consequently to the increase in energy requirement associated to pre-partum fetal growth and post-partum milk synthesis [3]. Negative energy balance leads to the extensive mobilization of adipose tissue, enhancing non-esterified fatty acids (NEFAs) and beta-hydroxybutyrate (BHB) concentrations in the bloodstream [4,5]. These physiological changes result in increased metabolic activity and production of reactive oxygen species (ROS) [6,7]. ROS can be defined as intracellular chemical species containing oxygen, including superoxide anion (O_2_^•−^) and hydrogen peroxide (H_2_O_2_) as well as hydroxyl radicals (^•^OH) [8]. These metabolites are intrinsic by-products of metabolism and play a crucial role in regulating normal cellular processes, including inflammation [9]. The organism is equipped with a complex system of endogenous and exogenous antioxidants, and the equilibrium between oxidants and antioxidants determines the individual’s redox balance [10]. Oxidant status occurs when the production of reactive oxygen species (ROS) surpasses the neutralization capacity of antioxidants, compromising cellular functions and damaging tissues [10,11]. Several studies have evaluated oxidant status in periparturient dairy goats, considering pro-oxidants and enzymatic and non-enzymatic antioxidant activity [3,12,13]. Abuelo et al. [14] previously demonstrated that the oxidative stress index (OSI), calculated as the ratio between the concentration of ROS and antioxidants (serum antioxidant capacity; SAC), provides a more accurate prediction of the oxidant status of transitioning dairy cattle compared to the assessment of pro-oxidants and antioxidants separately. These findings highlight the importance of evaluating not only their individual concentrations but also their relationship, as the imbalance between oxidants and antioxidants defines the concept of oxidative stress [14,15].

The overall goal of the study was to explore alterations in redox balance (ROS, SAC and OSi) during the peripartum period in dairy goats. We hypothesized that this physiological period would be characterized by significant changes in redox balance, with oxidative stress peaking around kidding. Additionally, we aimed to investigate the associations between oxidative biomarkers (ROS, SAC and OSi) and metabolic biomarkers (NEFAs and BHB) in order to understand the interplay between oxidative stress and metabolic changes during this critical phase of the reproductive cycle. Based on previous studies in dairy cattle [16,17], we hypothesized a potential positive correlation between oxidative (ROS, OSi and SAC) markers and metabolic (NEFAs and BHB) markers in transitioning dairy goats.

## 2. Materials and Methods

The trial was performed in northern Italy at the “La Quintalina” dairy goat farm (Anzano del Parco, CO, Italy). All animal procedures were carried out in accordance with EU Directive 2010/63/EU for animal experiments. Thirty-two secundiparous Alpine dairy goats (age: 23.13 ± 0.34 months; milk yield: 3.93 ± 1.23 L;) were housed in a free stall barn. The selected animals did not exhibit any health issues (e.g., metabolic, mastitis) on their previous lactation. The diet administrated during the whole trial is shown in Table 1. Samples of hay and concentrate were collected weekly and analyzed for dry matter (DM), crude proteins (CP), ether extract (EE), neutral detergent fibre (NDF), acid detergent fibre (ADF), lignin (ADL), starch, ash, calcium, phosphorus, digestable protein, forage unit for milk production (FMU), metabolizable energy (ME), milk net energy (ENL), dry matter (DM) digestability and organic matter (OM) digestability. Feed samples were dried and ground with a 1 mm screen according to the “Official Methods of Analysis” [18]. In brief, DM was determined by placing samples in pre-weighed aluminium bags and subsequently in a forced-air oven at 65 °C until constant weight. Crude proteins (CP) was determined using the Kjeldahl method by multiplying the nitrogen content by 6.25 (AOAC method 2001.11). Crude fiber (CF), NDF and ADF were determined placing the samples in filtering bags and analyzing them in an ANKOM Fiber Analyzer. Crude fuber (CF) was determined accoriding to AOACs Ba6a-05 method, while NDF, ADF and ADL were analyzed following the method of Van Soest et al. [19]. Neutral detergent fiber was determined using Neutral Detergent Solution (NDS) with α-amilase, ADF was determined using Acid Detergent Solution (ADS) and ADL was determined using solforic acid at 72%. Ether extract (EE) was extracted using ethyl ether in a Soxtec extractor (AOAC 2003.05). Total ash content was obtained after incineration at 550 °C for 3 h (AOAC method 942.05). Forage unit for milk production (FMU), metabolizable energy (ME), net energy for lactation (NEL), dry matter (DM) digestibility and organic matter (OM) digestibility were calculated by using the Small Ruminant Nutrition System (SRNS) software (Cornell Net Carbohydrate and Protein System; CNCPS).

Blood samples were collected from jugular vein into 9 mL evacuated free-anticoagulant tubes before the morning meal at dry-off (59.22 ± 9.47 days from partum; *n* = 32), kidding (*n* = 32) and 30 days in milk (DIM; *n* = 32). The entire group of goats was dried-off in December, the first blood sample was collected the same day and blood samples at kidding and 30 DIM were collected with a 1 day allowance from the exact date. Serum was obtained following centrifugation at 1300× *g* for 15 min of the blood samples, and it was frozen at −80 °C until further analysis. Total cholesterol was determined using an enzymatic immunoassay kit (Instrumentation Laboratory s.p.a, Milan, Italy). The analyses to determine triglycerides, NEFAs and BHB were performed using an automated spectrophotometer and RANDOX reagents (Randox, Crumlin, UK). Reactive oxygen species (ROS) were determined using the method of Trotti et al. [20] via a spectrophotometric d-ROM test (Diacron International, Grosseto, Italy). Results are expressed in arbitrary ‘Carratelli Units’ (CarrU), where 1 CarrU is equivalent to the oxidizing power of 0.08 mg H_2_O_2_/dL. Serum antioxidant capacity (SAC) was determined with an OXY-Adsorbent Test (Diacron International, Grosseto, Italy) [20]. Results are expressed as mmol HClO/mL. The oxidative stress index (OSi) was calculated as the ratio of ROS and SAC (ROS/SAC).

### Statistical Analysis

Data were analyzed with a repeated-measures model using a MIXED procedure in SAS 9.2 (SAS Inst., Inc., Cary, NC, USA). Sampling points were considered as fixed effects and goat was considered as the random effect. Data normality was assessed. We used a heterogeneous first-order autoregressive covariance structure, resulting in the smallest Akaike information criterion. The random error was assumed to be normally and independently distributed with zero expectation and common variance r. Pearson’s rho bivariate correlations of variables at sampling points were estimated by the PROC CORR statement in SAS. Values in the tables are presented as least squares means (±standard error of the mean; SEM). Significance was declared as *p* ≤ 0.05 and 0.01 and tendency as 0.1 < *p* < 0.05.

## 3. Results

### 3.1. Metabolic and Oxidative Biomarker Concentration in the Bloodstream

The concentration of metabolic and oxidative biomarkers in the bloodstream varied over time (Table 2). A graphical illustration of the trends of the biomarkers over time is available in the Supplementary Materials (Figure S1). The highest (*p* ≤ 0.01) concentration of triglycerides was observed at dry-off and followed a sharp decline the day of parturition, with triglyceride levels that remained stable up to 30 DIM. Serum total cholesterol concentration was lowest (*p* ≤ 0.01) at kidding in contrast with the concentration observed at dry-off and 30 DIM. BHB and NEFA concentrations during the first collection (dry-off) were significatively lower (*p* ≤ 0.01) than at parturition and 30 DIM. Furthermore, the highest (*p* ≤ 0.05) values of OSi and ROS were observed at kidding, while lower values were observed at dry-off and at 30 DIM. In contrast, SAC values did not exhibit a significant difference between the sampling points.

### 3.2. Pearson’s Rho Correlation Between Metabolic and Oxidative Biomarkers

Pearson’s rho correlation between metabolic and oxidative biomarkers is shown in Table 3. A significant positive correlation between triglycerides and cholesterol concentrations (rho:0.28; *p* ≤ 0.05) as well as between BHB and NEFA concentrations (rho:0.48; *p* ≤ 0.01) was observed. The oxidative stress index exhibited a moderate positive correlation (rho: 0.40; *p* ≤ 0.01) with ROS and a strong negative correlation with SAC (rho: −71; *p* ≤ 0.01). Finally, NEFAs tended to be correlated with ROS (rho: 0.19; *p* = 0.06) and were weakly correlated with OSi (rho:0.22; *p* < 0.05).

Pearson’s rho correlation between BHB and NEFAs at different timepoints is detailed in Table 4. A strong positive correlation between NEFAs at dry-off and BHB at kidding (rho: 0.56; *p* < 0.01) and 30 DIM (rho: 0.54; *p* < 0.01) was observed. Non-esterified fatty acids were strongly correlated with BHB at kidding (rho: 0.65; *p* < 0.0001) and moderately correlated 30 days after parturition (rho: 0.40; *p* < 0.05)

Pearson’s rho correlation between ROS, SAC and OSi during the different timepoints is detailed in Table 5.

A moderate positive correlation between ROS at kidding and OSi at parturition and 30 DIM was observed. A moderate negative correlation between ROS at kidding and SAC at 30 DIM was observed. Furthermore, SAC at dry-off, parturition and 30 days after kidding was negatively correlated with OSi at the same timepoints.

## 4. Discussion

The periparturient period in dairy animals represents a critical phase characterized by different physiological changes and is often related to metabolic stress [1]. During this time, dairy cattle may experience oxidative stress [15], which has been linked to metabolic diseases occurring in the peripartum period [21]. In the current study, we investigated the alterations in redox balance and the relationships between oxidative and metabolic biomarkers in peripartum dairy goats.

This study observed a decrease in serum triglycerides after kidding. This trend was similar to the results observed in dairy cows and may be associated with the uptake of triglycerides in the mammary gland for milk fat synthesis [22,23]. Low serum total cholesterol concentration at dry-off was probably related to the higher energy requirement for fetal growth and for steroid hormone synthesis [24], and the increase during lactation may be related to the intense adipose tissue mobilization [22].

The plasma concentration of NEFAs is related to the energy balance, and the gradual increase at kidding it is likely consequent to the intense adipose tissue mobilization that occurs during the transition period, while the decrease after kidding probably reflects the utilization of fatty acids in the mammary gland for milk fat synthesis [23]. These results were similar to the findings of Radin et al. [23], who observed a rise in NEFAs pre-partum and a drop 14 days post-partum in multiparous dairy goats. A lower BHB concentration at dry-off and an increase at kidding and early lactation was already observed in dairy animals and reflects the physiological rise in NEFA levels during the transition period [22,24,25]. Despite the increase in BHB concentration being linked with an NEB situation, the results of this study showed the concentration of this metabolite ranging from 0.3 to 0.5 mmol/L, far below the subclinical ketosis threshold of 1–1.4 mmol/L post-partum [26,27].

According to McCarthy et al. [28], Pilotto et al. [25] and Invernizzi et al. [16], there is an extremely weak relationship between fatty acid blood concentration and BHB during the periparturient period in dairy cows. In contrast, we observed a moderate correlation between NEFAs and BHB concentration that is corroborated by previous studies in dairy goats. Zamuner et al. [29] and Karagiannis et al. [30] reported a moderate-to-strong relationship between the concentration of NEFAs and BHB, suggesting a stronger association between these two markers in small ruminants than in dairy cows. In particular, we found a strong positive correlation between NEFAs at dry-off with BHB at kidding and 30 DIM. These correlations underline the importance of investigating free fatty acid blood concentrations before parturition in order to potentially prevent the development of subclinical ketosis during early lactation. Further analysis could provide valuable insights into the role of pre-partum NEFA concentrations in preventing and mitigating the risk of metabolic disorders in periparturient dairy goats.

Reactive oxygen species (ROS) are by-products of energy production through oxidative phosphorylation, and their rise indicates an increased energy demand in animals. Serum ROS concentration reached a peak during parturition in contrast to dry-off and 30 DIM. These results were consistent with findings observed by Invernizzi et al. [16] in dairy cows and could be explained by the peripartum stress that is related to the generation of lipid peroxides and ROS [31]. Bernabucci et al. [32] observed conflicting results showing a sharp decline pre-partum and a rise in ROS concentration post-partum in dairy cows. According to Miller et al. [21], the decrease in ROS around kidding should be related to the increase in antioxidant compounds characteristic of this physiological period. Celi et al. [13] observed similar results in dairy goats, showing a rise in ROS concentration from parturition to 2 weeks post-partum. Differences between these studies and our results could be related to the different number of animals involved and the choice of different physiological moments for sampling.

Serum antioxidant capacity (SAC) is an integrated parameter that reflects the combined activity of all antioxidants. Their concentration in plasma depends mainly on dietary intake and the synthetic capacity of specific organs, in particular the liver [33]. Contrary to our expectations, SAC values did not show a significant difference among the sampling points. According to the results obtained regarding serum ROS concentration, we expected a decrease in SAC concentration around parturition. In support of this hypothesis, studies carried out in dairy cows commonly report that the transition period is characterized by a depleted antioxidant status [17,32] due to the reduction in DMI and plasma concentration of nutritional antioxidants and co-factors, primarily utilized in late pregnancy and colostrum synthesis [34]. Our results contrast with those of Invernizzi et al.’s [16] study that demonstrated lower values of SAC at kidding than at dry-off and 30 DIM. In vitro [35] and in vivo [17] studies have observed that high NEFA and BHB concentrations decreased antioxidant levels in dairy cows. Huang et al. [3] reported that dairy goats with subclinical ketosis showed lower total antioxidant capacity (T-AOC) 1 week prepartum compared to healthy animals, suggesting that elevated NEFA and BHB levels may impair antioxidant capacity. However, T-AOC levels increased at parturition, returning to values comparable to those of healthy animals. In our experiment, despite the increase in NEFA and BHB concentrations at kidding, none of the animals exceeded the physiological thresholds antepartum (NEFAs ≥ 0.3–0.5 and BHB ≥ 0.6–0.8 mmol/L) and post-partum (NEFAs ≥ 0.7–1.0 and BHB ≥ 1.0–1.4 mmol/L) [27], and the sampling time differed (1 week prepartum vs. dry-off), which may explain why no reduction in antioxidant capacity was observed. Additionally, we observed a moderate negative correlation between ROS at parturition and SAC post-partum. This finding may suggest an implication of pro-oxidant concentrations at kidding on the antioxidant defense mechanisms in the first month of lactation.

The evaluation of oxidant status, comparing pro-oxidants and antioxidants through Osi, suggests that dairy goats may experience oxidative stress after parturition. The highest value of OSi observed at kidding and the lowest values observed at dry-off and early lactation were consistent with the results found by Invernizzi et al. [16] in dairy cows and reflected the significantly higher serum ROS concentration around parturition. The positive correlation between ROS and OSi at parturition and post-partum further indicates that the production of oxidants during kidding has detrimental effects on the oxidative status of the animal during the first month of lactation. Moreover, Zhang et al. [36] observed that early lactating dairy cows exhibiting high antioxidant ability rarely manifest ketosis. Supplementation of vitamins and certain trace minerals could have a positive effect in counteracting oxidative stress and preventing the development of some dairy ruminant diseases [11,37]. Additionally, the inclusion of alternative feed resources and by-products rich in polyphenols, tannins and antioxidants could be considered as dietary strategies to support dairy goats during critical physiological stages, enhancing their antioxidant capacity and overall resilience [38,39,40,41]. Bernabucci et al. [17] and Invernizzi et al. [16] observed a positive correlation between metabolic markers (BHB and NEFAs) and oxidative markers (ROS and ROM) in dairy cows, suggesting a potential involvement of metabolic markers in triggering oxidative stress. In the current study, NEFAs tended to be weakly correlated with ROS levels and were weakly correlated with OSi. These findings may be partially explained by the predisposition of NEFAs to be oxidation target molecules in the lipid peroxidation process and their ability to increase the metabolism of fatty acids through the activation of PPARs transcription factors. According to Sordillo et al. [9], peroxisomal β-oxidation is a significant source of ROS in dairy cows. An elevated NEFA concentration allows for the activation of PPARα [1] that binds with specific response elements (PRRE) of DNA, promoting the transcription of target genes involved in peroxisomal β-oxidation [42,43]. These findings suggest that NEFAs may be involved in the development of oxidant status, and, consequently, ROS and OSi could be considered as initial indicators to detect goats at risk of developing metabolic disorders. Subsequent confirmation of this assessment could be achieved through monitoring variations in NEFA concentrations.

Oxidative stress can increase susceptibility to metabolic disorders [44,45], leading to lower daily milk yield, decreasing fertility and increasing disease incidence. For this reason, oxidative biomarkers have been proposed as potential early indicators for predicting the onset of metabolic diseases in dairy cows [11,46]. Furthermore, Wisnieski et al. [47] observed that biomarkers of oxidant status predicted the development of metabolic diseases in a more accurate way compared to current biomarker monitoring methods, such as measuring NEFA and BHB levels between 7–10 days before and a few weeks after parturition. The results we observed could support the potential use of some oxidative biomarkers, in combination with NEFA detection, to identify goats at dry-off that are at risk of developing metabolic diseases. The identification of suitable biomarkers with their relative cut-off could provide additional time to implement targeted nutritional strategies, such as the supplementation of antioxidants, and management practices to reduce the incidence of diseases and improve the productivity and well-being of dairy goats.

## 5. Conclusions

In conclusion, the metabolic markers observed in the study align with previous findings in dairy animals during the transition period. The strong correlation between NEFAs at dry-off and BHB at kidding and 30 days post-partum highlights the potential influence of late-pregnancy NEFA concentrations on the metabolic status of dairy goats during early lactation. ROS and OSi showed the highest values at kidding, suggesting oxidative stress during this period, while SAC did not show significant variance across the designated time points. Although the correlation between metabolic markers (NEFAs) and oxidative indices (ROS and OSi) was weak, these findings suggest that NEFAs may contribute to the development of oxidant status in dairy goats. Furthermore, these results could support the use of oxidative biomarkers for the early identification of animals at risk of developing metabolic disorders. Early detection could allow for timely nutritional or management interventions, potentially reducing the incidence of metabolic disorders within the herd. Further research is needed to determine the reliability of oxidative biomarkers in predicting metabolic status and their integration into routine monitoring practices.

## Figures and Tables

**Table 1:** Ingredients and chemical composition of the diets (% on a DM basis) used during the trial.

Ingredients	Dry-Off	Kidding	Lactation
Ryegrass hay	100		
Mixed hay ^1^		76.6	45.7
Concentrate ^2^		23.4	27.4
Alfalfa hay			15.2
Whole corn			11.7
Tot	100	100	100
Dry matter	88.5	88.2	88.1
Crude protein	10.1	12.2	14.2
Ether extract	1.7	2.9	3.2
Crude fiber	30.5	31.1	24.6
NDF	59.6	56.2	43.3
ADF	36.5	31.6	24.8
ADL	5.8	4.3	3.6
Calcium	0.5	0.9	0.8
Phosporus	0.3	0.3	0.4
Starch	-	10.7	20.5
Ash	8.5	6.28	5.3
NFCs ^3^	20.1	22.4	52.7
Digestible protein	6.02	8.45	10.11
ME (Mcal/kg DM)	2.10	2.38	2.57
NEL (Mcal/kg DM)	1.35	1.53	1.66
FMU (unit/kg DM) ^4^	0.79	0.90	0.98
DM digestibility (%)	60.13	65.80	70.76
OM digestibility (%)	62.93	68.49	73.68

^1^ From a multi-species meadow. ^2^ Commercial concentrate composed of corn 19%, wheat flour 16%, soybean meal 16.3%, barley 13%, wheat bran 13%, corn cake 12.2%, sunflower meal 6%, molasses, calcium carbonate, dicalcium phosphate and NaCl; CP 22%, LG 6%, CF 8%. ^3^ Non-fiber carbohydrates (NFCs) calculated as 100 − (CP + ether extract + ash + NDF). ^4^ Forage unit for milk production (FMU; 1FMU = 1700 Kcal or 7.12 MJ) calculated as NEL (Kcal/kg DM)/1700.

**Table 2:** Average values of total cholesterol, triglycerides, NEFA, BHB, ROS, SAC and OSi. Significance was declared with * *p* ≤ 0.05 and ** *p* ≤ 0.01 within each row.

	Dry-Off LSM ± SEM	Kidding LSM ± SEM	30 DIM LSM ± SEM
Total cholesterol (mmol/L)	2.72 ± 0.10	1.98 ± 0.10 **	2.58 ± 0.10
Triglycerides (mmol/L)	0.32 ± 0.01 **	0.15 ± 0.01	0.18 ± 0.01
NEFAs (mmol/L)	0.21 ± 0.04	0.55 ± 0.04 **	0.32 ± 0.04
BHB (mmol/L)	0.33 ± 0.02	0.44 ± 0.02 **	0.32 ± 0.02
ROS (Carr. U)	160.91 ± 6.99	183.13 ± 6.99 *	174.36 ± 6.99
SAC (umol HClO/mL)	433.81 ± 28.89	434.87 ± 28.89	450.78 ± 28.89
OSi (Carr. U/(umol HCLO/mL)	0.40 ± 0.03	0.50 ± 0.03 *	0.42 ± 0.03

NEFAs: non-esterified fatty acids; BHB: β-hydroxybutyrate; ROS: reactive oxygen species; SAC: serum antioxidant capacity; OSi: oxidative stress index. SEM: standard error of the mean.

**Table 3:** Pearson’s rho correlations between NEFAs, BHB, total cholesterol, triglycerides, ROS, SAC and OSi. Significance was declared with * *p* ≤ 0.05 and ** *p* ≤ 0.01 (*n* = 32) within each row.

		NEFA	BHB	TC	TRI	ROS	SAC	OSi
NEFAs	Rho	1	0.48	−0.15	−0.26	0.19	0.11	0.22
	*p* value	_	<0.0001 **	0.16	0.01 *	0.06	0.26	0.03 *
BHB	Rho		1	0.17	−0.14	0.10	0.17	0.11
	*p* value		_	0.09	0.16	0.33	0.09	0.29
TC	Rho			1	0.28	−0.06	0.08	−0.18
	*p* value			_	0.005 *	0.55	0.44	0.08
TRI	Rho				1	0.20	0.03	0.04
	*p* value				_	0.05	0.79	0.71
ROS	Rho					1	0.04	0.40
	*p* value					_	0.72	<0.0001 **
SAC	Rho						1	−0.71
	*p* value						_	<0.0001 **
OSi								1
								_

NEFAs: non-esterified fatty acids; BHB: β-hydroxybutyrate; TC: total cholesterol; TRI: triglycerides; ROS: reactive oxygen species; SAC: serum antioxidant capacity; OSi: oxidative stress index.

**Table 4:** Pearson’s rho correlations between NEFAs and BHB at dry-off, kidding and 30 DIM. Significance was declared with * *p* ≤ 0.05 and ** *p* ≤ 0.01 (*n* = 32) within each row.

			Dry-Off		Kidding		30 Days in Milk	
			NEFA	BHB	NEFA	BHB	NEFA	BHB
Dry-off	NEFA	Rho	1	−0.29	0.06	0.56 **	0.26	0.54 **
		*p* value	_	0.11	0.76	0.001	0.15	0.001
	BHB	Rho		1	−0.005	−0.19	−0.09	0.04
		*p* value		_	0.98	0.31	0.60	0.83
Kidding	NEFA	Rho			1	0.65 **	−0.14	0.27
		*p* value			_	<0.0001	0.42	0.13
	BHB	Rho				1	−0.13	0.32
		*p* value				_	0.46	0.07
30 days in milk	NEFA	Rho					1	0.40 *
		*p* value					_	0.02
	BHB	Rho						1
		*p* value						_

**Table 5:** Pearson’s rho correlations between ROS, SAC and OSi at dry-off, kidding and 30 DIM. Significance was declared with * *p* ≤ 0.05 and ** *p* ≤ 0.01 (*n* = 32) within each row.

			Dry-Off			Kidding			30 Days in Milk		
			ROS	SAC	OSi	ROS	SAC	OSi	ROS	SAC	OSi
Dry-off	ROS	Rho	1	0.19	0.40 *	0.06	0.17	−0.19	0.44 *	0.24	0.05
		*p* value	_	0.29	0.02	0.75	0.36	0.30	0.01	0.18	0.76
	SAC	Rho		1	−0.72 **	−0.004	0.10	−0.53 **	0.24	0.45 *	−0.24
		*p* value		_	<0.0001	0.98	0.33	0.002	0.19	0.01	0.19
	OSi	Rho			1	0.16	−0.50 **	0.39 *	0.17	−0.36 *	0.43 *
		*p* value			_	0.38	0.004	0.03	0.36	0.045	0.01
Kidding	ROS	Rho				1	0.26	0.42 *	0.46	−0.48 **	0.52 **
		*p* value				_	0.15	0.02	0.01	0.005	0.002
	SAC	Rho					1	−0.76 **	0.16	0.71	−0.40
		*p* value					_	<0.0001	0.38	<0.0001	0.02
	OSi	Rho						1	0.05	−0.52 **	0.41
		*p* value						_	0.78	0.002	0.02
30 days in milk	ROS	Rho							1	0.11	0.46 **
		*p* value							_	0.53	0.008
	SAC	Rho								1	−0.72 **
		*p* value								_	<0.0001
	OSi	Rho									1
		*p* value									_

ROS: reactive oxygen species; SAC: serum antioxidant capacity; OSi: oxidative stress index.

## Data Availability

The original contributions presented in this study are included in the article/Supplementary Material. Further inquiries can be directed to the corresponding author.

## Supplementary Materials

The following supporting information can be downloaded at: https://www.mdpi.com/article/10.3390/metabo15120790/s1, Figure S1: Average values of total cholesterol, triglycerides, NEFAs, BHB, ROS, SAC and OSi.
