# Supplementary material for: Association of Oxidative Stress Biomarkers with Metabolic Parameters in Dairy Goats During the Periparturient Period
Source: Metabolites. 2025 Dec 11;15(12):790. doi: 10.3390/metabo15120790 (PMC12734880; doi:10.3390/metabo15120790)

Metabolites- Supplementary materials

Association of oxidative stress biomarkers with metabolic parameters in dairy goats during the periparturient period

Giovanna Meli*, Valentina Fumo, Wenning Chen, Giovanni Savoini, Guido Invernizzi

Dipartimento di Medicina Veterinaria e Scienze Animali, Università degli studi di Milano, Via dell'Università 6, Lodi, Italy

Figure S1: Average values of total cholesterol, triglycerides, NEFA, BHB, ROS, SAC and OSi. Significance was declared with *p ≤ 0.05 **p ≤ 0.01

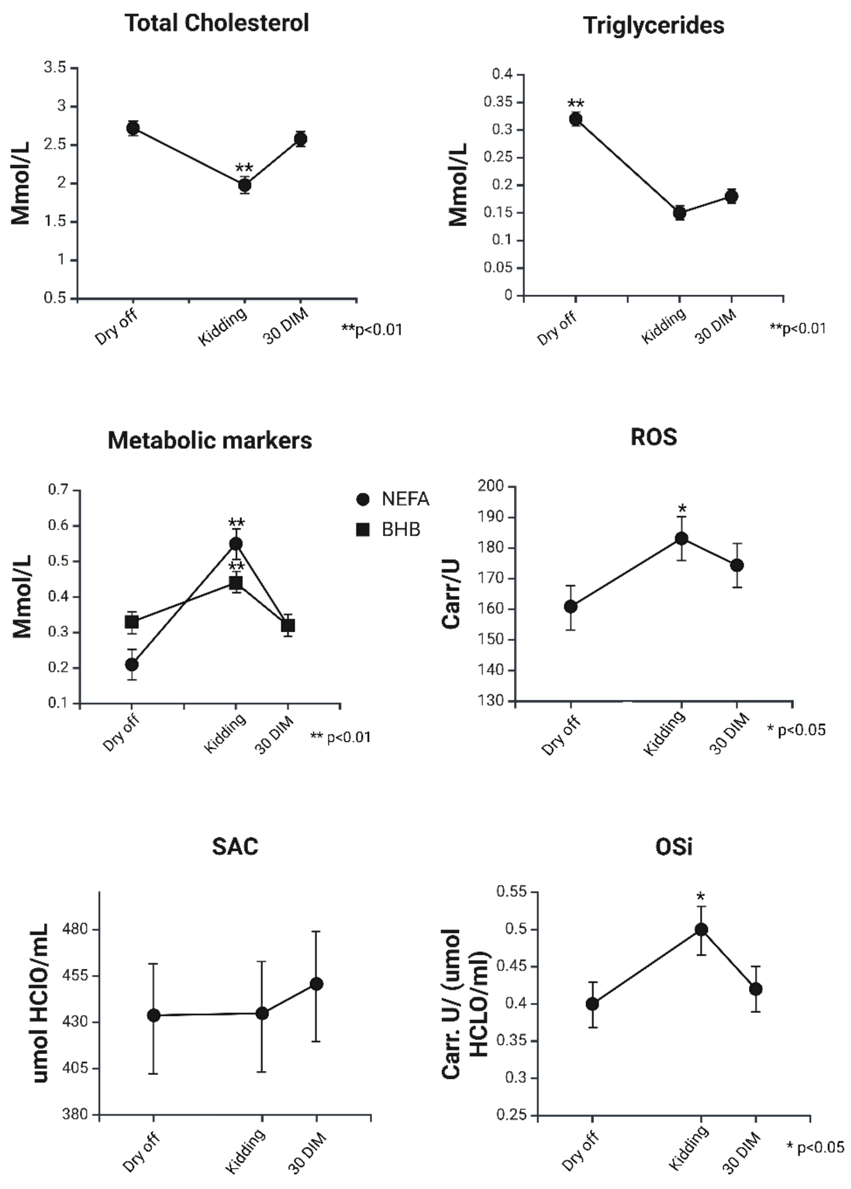

Supplement: Supplementary file 1 [file metabolites-15-00790-s001.zip › metabolites-3431763-supplementary.pdf]
